# An Assessment of the In Vitro Antioxidant Activity of Cobalt Nanoparticles Synthesized From Millettia pinnata, Butea monosperma, and Madhuca indica Extracts: A Comparative Study

**DOI:** 10.7759/cureus.59112

**Published:** 2024-04-26

**Authors:** Iadalin Ryntathiang, Archana Behera, Titus Richard, Mukesh Kumar Dharmalingam Jothinathan

**Affiliations:** 1 Centre for Global Health Research, Saveetha Medical College and Hospitals, Saveetha Institute of Medical and Technical Sciences, Chennai, IND; 2 Department of English, Saveetha School of Engineering, Saveetha Institute of Medical and Technical Sciences, Chennai, IND

**Keywords:** dpph, cobalt nanoparticles, green synthesis, antioxidant activity, nanotechnology

## Abstract

Objective

This study aimed to synthesize cobalt nanoparticles (CoNPs) via the green synthesis method using *Millettia pinnata (M. pinnata)*, leaf (MPL), *Butea monosperma (B. monosperma) *flower (BMF), and *Madhuca indica (M. indica) *flower (MIF) as eco-friendly reducing agents. It further aimed to compare the effectiveness of these plant extracts in CoNPs production and evaluate the antioxidant activities of the synthesized nanoparticles (NPs), establishing a link between the phytochemical constituents of the extracts and the antioxidant capacity of CoNPs for potential applications in drug development and environmental sustainability.

Materials and methods

CoNPs were synthesized using aqueous extracts of MPL, BMF, and MIF. These extracts act as stabilizing and self-reducing agents. Initially, the presence of CoNPs was detected visually by observing a color change. To confirm this observation, UV-visible spectroscopy and Fourier transform infrared (FTIR) spectroscopy were employed. UV-visible spectroscopy helps in analyzing the absorption of light by the CoNPs, while FTIR spectroscopy is used to identify the functional groups present in the NPs. Subsequently, the antioxidant activity of the synthesized CoNPs was assessed using the 1,1-diphenyl-2-picryl hydroxyl (DPPH) radical-scavenging assay. This assay measures the ability of antioxidants to neutralize free radicals by determining the reduction in the DPPH radical's absorption. To ensure the reliability of the results, the experiments were conducted in triplicate. Statistical analysis was then performed to compare the antioxidant effectiveness of the different plant extracts used in synthesizing the CoNPs. This analysis helps in determining any significant differences in antioxidant activity among the extracts.

Results

UV-visible spectral analysis confirmed the successful synthesis of CoNPs, revealing characteristic absorption peaks. For *M. pinnata* leaf extract (MPLE), the maximum peak was observed at ~272 nm, while *B. monosperma* flower extract (BMFE) exhibited a peak at ~276 nm, and *M. indica* flower extract (MIFE) revealed a maximum peak at ~320 nm. FTIR analysis further validated the presence of organic molecules from plant components on the outer layer of CoNPs, indicating successful capping and stabilization by phytochemicals from the extracts. The spectra displayed various peaks at different wavenumbers: MPLE showed prominent peaks at 3335 cm^−1^, BMFE showed distinct peaks at 3314 cm^−1^, and MIFE exhibited significant peaks at 3261 cm^−1^. Among the three types of CoNPs tested, those synthesized using MIFE exhibited the highest inhibition of 87.67% at a concentration of 60 µL. This higher inhibition was compared to those synthesized using BMFE and MPLE. This study suggests that the CoNPs synthesized on MIFE can serve as an antioxidant agent because of their remarkable free radical-scavenging activity.

Conclusions

The study highlights the potential of CoNPs synthesized using MIFE as they exhibited superior antioxidant activity compared to those synthesized with BMFE and MPLE. Therefore, the study underscores the promise of MIFE as a valuable natural resource for producing CoNPs abundant in antioxidants. Furthermore, it emphasizes the importance of implementing environmentally friendly synthesis techniques to produce nanomaterials that are both safe for the environment and biologically effective.

## Introduction

Nanotechnology involves designing, characterizing, and producing structures at the nanometer scale, where materials exhibit unique characteristics compared with larger dimensions [1]. Nanotechnology has revolutionized various industries by producing and applying nanoparticles (NPs) in fields such as biomedical devices, engineering, food, medicine, and agriculture [2]. Among them, metal NPs, such as palladium, titanium oxide, strontium, platinum, selenium, zirconium dioxide, silver, cerium oxide, gold, silver, sulfide, zinc oxide, chitosan, cellulose, silica, iron, cobalt, and copper, have shown promise in biomedical applications because of their antibacterial and antioxidant properties and ability to change the oxidation state [3-5].

However, cobalt nanoparticles (CoNPs) are highly promising because of their exceptional magnetic, electrical, and catalytic properties, attracting interest from scientists across various fields for applications such as catalysis, magnetic composites, sensors, and memory [6]. Traditional NP production methods have certain drawbacks like high costs and toxic byproducts. Hence, scientists are now seeking eco-friendly and cost-effective approaches, such as those using plant extracts containing bioactive compounds or exploring biogenesis for diverse NP synthesis [7]. Plants can produce NPs because of their redox activities, which makes them more suitable for chemical and physical processes. A simple yet reliable process can be used to produce plant extracts that are nontoxic and easy to handle [8].

*Millettia pinnata (M. pinnata)*, a member of the Fabaceae family, comprises over 200 plant species grown globally in tropical and subtropical climates. Pongamia, also part of Fabaceae, was previously known as a significant group of flowering plants, with Millettia having 363 species names listed, of which 202 are recognized [9]. Millettia species are traditionally used for treating various ailments like rheumatoid arthritis, amenorrhea, tuberculosis, joint pain, and tumors, and as insecticides, pesticides, antiseptics, blood purifiers, and wound-healing agents [10-11]. The plant possesses anti-inflammatory, anti-proliferative, antioxidant, and anticancer properties because of its diverse phytochemical components, such as glycosides, flavonoids, furane flavones, chromonoflavones, and furanodiketones [10,12].

Similarly, the plant *Butea monosperma (B. monosperma)* (Lam) Taub (Butea frondosa) is a member of the Fabaceae family, also referred to as Palas in Sanskrit, and has been traditionally used as a medicine. Flowers, leaves, bark, and seeds are purgative, ophthalmic, anthelmintic, depurative, and tonic [13-14]. *B. monosperma* extracts exhibit several in vivo and in vitro properties, such as hepatoprotective, antitumoral, antihyperglycemic, and wound healing [15]. According to Munawar et al. [16], *B. monosperma* stem ethanolic extracts have significantly more antioxidant activity than flower extracts. A previous study has found that *B. monosperma* has significant potential as a source of valuable chemicals with notable qualities, such as enzyme inhibition and antioxidant activity [17].

*Madhuca indica (M. indica)*, commonly referred to as Mahua, is a prominent economic plant that grows in the subtropical region of the Indo-Pakistan peninsula. It is a member of the Sapotaceae family. This plant has been shown to contain a variety of phytochemicals, including terpenoids, cardiac glycosides, proteins, lipids, anthraquinone, coumarin, carbohydrates, flavonoids, alkaloids, and tannins. This plant is used in several forms as an astringent, heating agent, emollient, demulcent, and stimulant. Various parts of this plant have their own benefits [18]. The bark, leaves, and flowers of Mahua have a variety of medicinal benefits, including antipyretic, anticancer, antidiabetic, antimicrobial, cytotoxic, anti-inflammatory, antiepileptic, antinociceptive, antidiarrheal, anti-inflammatory, anti-ulcer, and hepatoprotective properties [19]. A previous study found that *M. indicia'*s methanolic extract has strong antioxidant properties. The antioxidant property of the methanolic extract of *M. indica* may be attributed to the presence of several phenolic components. These initial findings lend support to the traditional medicinal use of the plant as an antioxidant [20].

The objective of this research is to evaluate and compare the antioxidant activity of CoNPs mediated by *M. pinnata* leaf extract (MPLE), *B. monosperma* flower extract (BMFE), and *M. indica* flower extract (MIFE). This study aims to offer an essential and comparative assessment based on research on phytochemistry and biological activity. These findings have great potential for sustainable and environmentally friendly biomedical applications and hold promise for developing innovative antioxidant alternatives.

## Materials and methods

Collection of materials

Fresh leaves of the Indian plant *M. pinnata*, popularly known as Pongamia, were collected from Chennai, Tamil Nadu, India. *B. monosperma* and *M. indica* flowers were collected from Balasore, Orissa, India. The samples were authenticated by the Centre for Advanced Studies in Botany at the University of Madras, Chennai, India.

Preparation of aqueous extract

MPL, BMF, and MIF were washed with running water and subsequently rinsed with distilled water, followed by dicing into small fragments and shade drying for three days. Afterward, they were mechanically ground into a powder using a blender. Approximately 10 g of each dried MPL, BMF, and MIF powder was separately mixed with 100 mL of distilled water in a conical flask. The mixtures were heated to 60 °C for 20 minutes using a heating mantle while continuously stirring. After cooling, the liquid was filtered through muslin fabric to remove debris, and the filtrate was further purified by filtration through Whatman No. 1 filter paper to eliminate any remaining particles. The resulting aqueous extract was collected and stored at -4 °C in a refrigerator for future analysis [21].

Preparation of cobalt chloride

A standard stock solution of cobalt chloride (CoCl_2_) (1 mM) was prepared by dissolving it in 300 mL of distilled H_2_O.

Synthesis preparation of cobalt nanoparticles

For CoNPs synthesis, about 10 mL of MPLE, BMFE, and MIFE were individually added to a 90 mL solution containing CoCl_2_ (1 mM) and agitated at 35°C. The reaction mixture was consistently monitored and recorded. The mixtures were then incubated in a dark environment on a rotary shaker at 300 rpm for 3 hours. A visual examination of the mixture's color was conducted. The prepared mixture was incubated at room temperature for 72 hours. After the incubation period, the mixture exhibited a color change. The color change indicates the nanoparticle synthesis. The control exhibits no color change upon the addition of CoCl_2_.

Characterization of cobalt nanoparticles

Using a Labman Scientific UV-visible spectrophotometer, after 24 hours, the UV-visible spectra of the CoNPs were observed at 200-400 nm (wavelength). A Fourier transform infrared (FTIR) spectrometer was employed to perform the measurement over the 400-4000 cm^-1^ range. The different functional groups present in the samples (MPLE, BMFE, and MIFE) were documented.

Antioxidant activity (DPPH)

The antioxidant activity of CoNPs synthesized by MPLE, BMFE, and MIFE was assessed using the 1,1-diphenyl-2-picryl hydroxyl (DPPH) free radical scavenging method. This experiment aimed to evaluate the antioxidant activity of various concentrations of MPLE, BMFE, and MIFE, as well as standard ascorbic acid, against DPPH free radicals. This assay followed the methodology outlined by Behera et al. [22].

## Results

**Figure 1:**
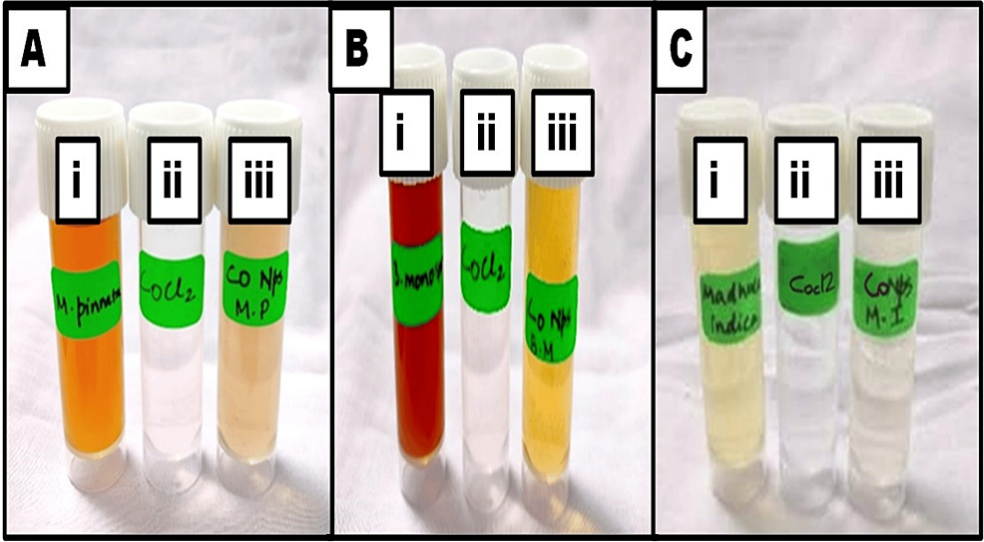
Visual observation of cobalt nanoparticles: [A] MPLE, [B] BMFE, and [C] MIFE MPLE: *Millettia pinnata *leaf extract; BMFE: *Butea monosperma* flower extract; MIFE: *Madhuca indica* flower extract; CoCl_2_: cobalt chloride

The successful production of CoNPs via a biological approach was observed, with the confirmation of nanoparticle formation shown by color changes, as depicted in Figures 1A-1C. Initially, the MPLE extract exhibited an orange color (Figure 1A (i)), while the metal precursors appeared colorless (Figure 1A (ii)). After the reaction was complete, the solution turned light brown, as depicted in Figure 1A (iii). Similarly, the BMFE extract initially appeared dark brown, as shown in Figure 1B (i), whereas the metal precursors are visible as colorless [Figure 1B (ii)]. Once the reaction was completed, the solution turned yellow, as shown in Figure 1B (iii). Likewise, the MIFE extract exhibited a light brown color [Figure 1C (i)], whereas the metal precursors appear colorless [Figure 1C (ii)]. After the completion of the reaction, the solution turned light gray, as depicted in Figure 1C (iii). CoNPs were produced in a simple and sustainable manner by using this technique. Throughout the synthesis, MPLE, BMFE, and MIFE play important roles as stabilizing and reducing agents.

Characterization of CoNPs

Uv-Visible Spectroscopy

**Figure 2:**
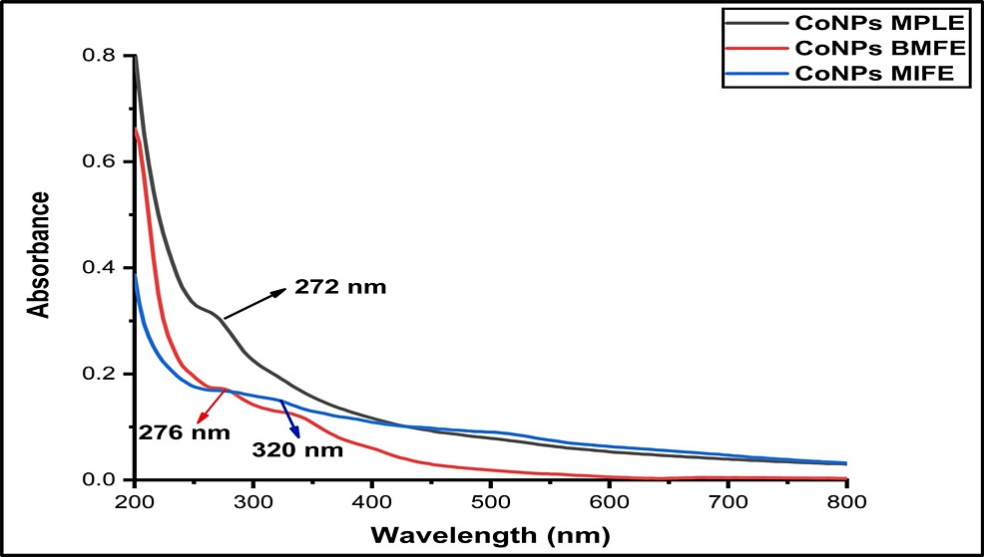
Ultraviolet-visible spectrum of cobalt nanoparticles synthesized from plant extracts MPLE: *Millettia pinnata* leaf extract (~272 nm); BMFE: *Butea monosperma* flower extract (~276 nm); MIFE: *Madhuca indica* flower extract (~320 nm)

This study involved screening plant extracts with medicinal properties and found that MPLE exhibited the desired ability to form CoNPs. The confirmation was obtained by evaluating the UV-visible spectra, which exhibited a peak absorption at ~272 nm, suggesting the synthesis of CoNPs during overnight incubation. Similarly, the BMFE also showed this capability, as demonstrated by the UV-visible spectra, which showed an absorption peak ranging from ~400 to 500 nm. This peak confirmed the d-d transition of the metal precursor. Additionally, there was a peak at around ~276 nm, indicating the sp3 hybridization of CoNPs. In addition, MIFE demonstrated the expected distinctiveness, as confirmed by UV-visible spectral analysis, which revealed a maximum absorption at ~320 nm after overnight incubation. This indicates that there was an interaction between the metal and the extract, resulting in the synthesis of CoNPs (Figure 2).

FTIR

**Figure 3:**
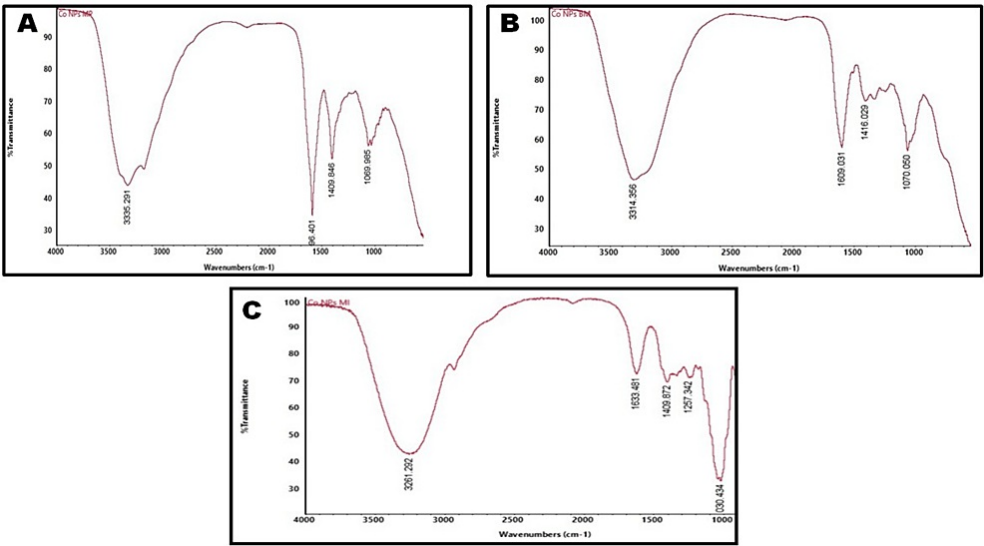
FTIR spectrum of cobalt nanoparticles synthesized using [A] MPLE, [B] BMFE, and [C] MIFE MPLE: *Millettia pinnata* leaf extract; BMFE: *Butea monosperma* flower extract; MIFE: *Madhuca indica* flower extract

FTIR spectroscopy is a common technique used to determine functional groups based on their specific wavenumbers ranging between 400 and 4,000 cm^−1^. In natural product research, this spectroscopic method is sufficient for identifying bioactive compounds. The spectra displayed various peaks at different wavenumbers in the CoNPs using MPLE. Figure 3A shows prominent peaks at 3335 cm−^1^. The significant peak at 3335 cm^−1^ represents N-H stretching, and the distinct peak at 1596 cm^−1^ corresponds to N-H bending. The peak at 1409 cm^−1^ signifies C-H bending, whereas the peak at 1069 cm^−1 ^indicates S=O stretching. Phytochemicals such as flavonoids, alkaloids, steroids, terpenoids, and phenols were abundant in MPLE.

Similarly, the FTIR spectrum provides details about the functional groups in the CoNPs synthesized using BMFE. The FTIR spectra showed distinct peaks at various wavenumbers, with a significant peak at 3314 cm^−1^ (Figure 3B). The prominent signal at 3314 cm^−1^ represents N-H stretching vibrations, suggesting the existence of water molecules absorbed from the plant extract. A notable peak at 1609 cm^−1^ represents C=C stretching (α, β-unsaturated ketone), whereas peaks at 1416 cm^−1^ and 1070 cm^−1^ suggest S-H bending and S=O bending, respectively. CoNPs demonstrate interactions with phenolic compounds, alkenes, amines, terpenoids, and flavonoids.

Similarly, FTIR analysis was conducted to synthesize CoNPs using MIFE as a capping agent. The analysis identified stable synthesized CoNPs that exhibited significant peaks at 3261 cm^−1^ as illustrated in Figure 3C. The peak at 3261 cm^−1^ represents O-H stretching vibrations, and the peak at 1633 cm^−1^ indicates C=C stretching. The peak at 1409 cm^−1^ signifies C-H bending, whereas the peak at 1030 cm^−1^ signifies C-N stretching.

Antioxidant activity

DPPH Assay

**Figure 4:**
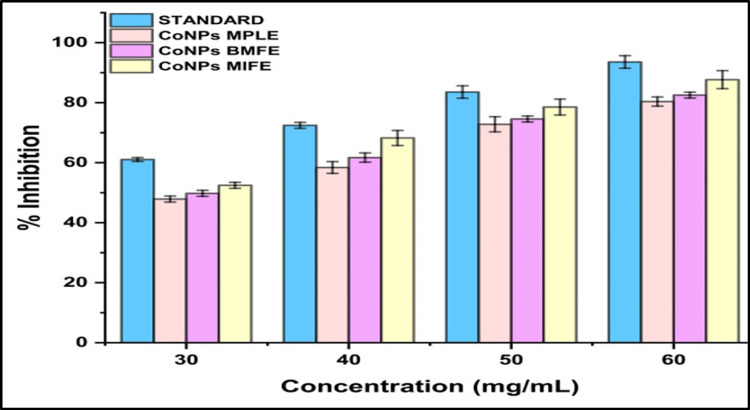
Antioxidant assay using DPPH of cobalt nanoparticles synthesized by plant extracts MPLE: *Millettia pinnata* leaf extract; BMFE: *Butea monosperma* flower extract; MIFE: *Madhuca indica* flower extract

A comparison of the antioxidant activity of three types of CoNPs with the standard (ascorbic acid) against DPPH radicals using free radical scavenging activity was performed. At a concentration of 60 µL, the standard showed 93.57% inhibition, MPLE showed 80.38% inhibition, BMFE showed 82.53% inhibition, and MIFE demonstrated 87.67% inhibition. Similarly, at a concentration of 30 µL, the standard exhibited 61.06% inhibition, whereas MPLE, BMFE, and MIFE exhibited 47.88%, 47.78%, and 52.46% inhibition, respectively (Figure 4).

## Discussion

This study compared the antioxidant activity of CoNPs synthesized using a green synthesis method involving MPLE, BMFE, and MIFE. This study involved the synthesis of CoNPs, and their characterization using visual observation, UV-visible spectrophotometry, and FTIR. The results were absorbed and recorded. The absorbance peak values were absorbed at ~272 nm (MPLE), ~276 nm (BMFE), and ~320 nm (MIFE). Antioxidant activity was assessed using the DPPH assay. This research seeks to make substantial contributions in the area of environmental nanomaterials and their applications in therapeutics.

The functional groups of CoNPs synthesized from MPLE, as revealed by FTIR analysis, were absorbed at wavelengths corresponding to 3335, 1596, 1409, and 1069 cm^−1^, indicating N-H stretching, N-H bending, C-H bending, and S=O stretching, respectively. Similarly, CoNPs derived from BMFE exhibited absorbance peaks at 3314, 1609, 1416, and 1070 cm^−1^, corresponding to N-H stretching, C=C stretching, S-H, and S=O bending, respectively, as observed by FTIR analysis. Likewise, CoNPs synthesized from MIFE showed absorption peaks at 3261, 1633, 1409, and 1030 cm^−1^, indicating various functional groups such as O-H, C=C, C-H, and C-N. The aqueous extracts of MPLE, BMFE, and MIFE contain essential phytochemicals, including flavonoids, alkaloids, steroids, terpenoids, and phenols [9,15,19].

The synthesis of metal NPs often involves a complex interplay between the biomolecules present in the plant extract and the metal ions, leading to distinctive optical and spectroscopic signatures. For instance, in cobalt oxide NPs synthesized from plant nutrients, the color change is caused by the interaction between conduction electrons in the NPs and incoming photons, as indicated by the presence of a distinct peak at around ~269 nm in the UV-visible spectrum. FTIR examination revealed characteristic peaks at 3261 cm^−1^ and 1636 cm^−1^, indicating O-H stretching and amides, respectively. These plant-derived molecules of the NPs play crucial roles in stabilization, capping, and reduction, as evidenced by the additional peaks at 1382 cm^−1^ and 1639 cm^−1^. Furthermore, the involvement of carbon and OH molecules enhances the reduction process and activity of Co₃O₄ NPs. This underscores the significance of plant-based synthesis methods in nanomaterial production [23].

When we compared the properties of CoNPs synthesized from all three extracts, MIFE exhibited a remarkable antioxidant capacity that surpasses that of BMFE and MPLE. The superior antioxidant activity of MIFE is due to its abundant phytochemical composition, which consists of flavonoids, saponins, and glycosidic substances. These bioactive compounds are recognized for their effectiveness in scavenging harmful free radicals, thereby offering protection against oxidative stress. MIFE consistently shows higher radical-scavenging activity, which indicates its potent antioxidant properties. The flavonoids present in MIFE, in particular, contribute significantly to this activity by stabilizing free radicals and preventing further oxidative damage [24-25]. In contrast, BMFE and MPLE, while also containing antioxidant phytochemicals, do not demonstrate the same level of activity. This could be due to lower concentrations or different types of phytochemicals that may not be as effective in neutralizing free radicals [26-28]. Additionally, one study [29] has reported that iron oxide nanoparticles (FeONPs) derived from *M. indica* showed the highest antioxidant activity at a concentration of 1000 μg/mL, with a value of 81.5% compared with plant extract and control, further emphasizing the potent antioxidant potential of MIFE.

Limitations

In this study, in vitro analyses were conducted to evaluate the antioxidant activity. However, additional In vivo research, including animal and clinical trials, would be beneficial for a better understanding of its effects.

## Conclusions

While each of the three plants exhibits unique medicinal properties and inhibitory activity, MIFE stands out for its exceptional antioxidant properties among the CoNPs synthesized (MPLE and BMFE). Various studies have confirmed *M. indica*’s potential as a rich source of antioxidants, with implications for health supplements and functional foods. Its superior antioxidant capacity holds promise for nutraceutical and pharmacological applications. Nonetheless, further comprehensive research is necessary to fully elucidate the mechanisms underlying these differences. *M. indica* has emerged as a frontrunner in this field, offering a valuable resource for combating oxidative stress and improving human health.

## References

[1] Mohandes A, Aghamaali MR, Sabouri Z, Darroudi M (2023). Biosynthesis of cobalt oxide nanoparticles (Co3O4-NPs) using Caccinia Macranthera extract and evaluation of their cytotoxicity and photocatalytic activity. Mater Sci and Eng: B.

[2] Iqbal J, Abbasi BA, Batool R (2019). Biogenic synthesis of green and cost-effective cobalt oxide nanoparticles using Geranium Wallichianum leaves extract and evaluation of in vitro antioxidant, antimicrobial, cytotoxic and enzyme inhibition properties. Mater Res Express.

[3] Rajeshkumar S, Bharath LV (2017). Mechanism of plant-mediated synthesis of silver nanoparticles - a review on biomolecules involved, characterisation and antibacterial activity. Chem Biol Interact.

[4] He X, Xue J, Shi L (2022). Recent antioxidative nanomaterials toward wound dressing and disease treatment via ROS scavenging. Mater Today Nano.

[5] Issaabadi Z, Nasrollahzadeh M, Sajadi SM (2017). Green synthesis of the copper nanoparticles supported on bentonite and investigation of its catalytic activity. J Clean Prod.

[6] Ali H, Yadav YK, Ali D, Kumar G, Alarifi S (2023). Biosynthesis and characterization of cobalt nanoparticles using combination of different plants and their antimicrobial activity. Biosci Rep.

[7] Hafeez M, Shaheen R, Akram B (2020). Green synthesis of cobalt oxide nanoparticles for potential biological applications. Mater Res Express.

[8] Bibi I, Nazar N, Iqbal M (2017). Green and eco-friendly synthesis of cobalt-oxide nanoparticle: characterization and photo-catalytic activity. Adv Powder Technol.

[9] Chen K, Tang H, Zheng L (2018). Identification of compounds with cytotoxic activity from Millettia dorwardi Coll. Et. Hemsl. Phytochem Lett.

[10] Rajeshkumar S (2016). Synthesis of silver nanoparticles using fresh bark of Pongamia pinnata and characterization of its antibacterial activity against gram positive and gram negative pathogens. Resour Eff Technol.

[11] Marco M, Deyou T, Gruhonjic A (2017). Pterocarpans and isoflavones from the root bark of Millettia micans and of Millettia dura. Phytochem Lett.

[12] Banoth RK, Shaik S, Dumpala T (2020). Evaluation of phytoconstituents, FT-IR analysis, total phenolic, flavonoid contents, in-vitro antibacterial and antioxidant studies of ethanolic root extract of Millettia pinnata. Int J Pharm Sci Res.

[13] Gadekar GP, Ghaoshal KP, Ghatole AM (2021). Butea monosperma bark extract to its green synthesis of silver nanoparticles and their antioxidant, total flavonoid content and antimicrobial activities. J Appl Biol Sci.

[14] Ghoshal KP, Gadekar GP (2020). Green nanoparticles from butea monosperma and evaluation of their antimicrobial activity. Int J Appl Chem Biol Sci.

[15] Subramaniyan B, Polachi N, Mathan G (2016). Isocoreopsin: An active constituent of n-butanol extract of Butea monosperma flowers against colorectal cancer (CRC). J Pharm Anal.

[16] Munawar T, Aruna K, Rao RS (2018). Evaluation of antibacterial and antioxidant activity of ethanolic extracts of butea monosperma. World J Pharm Res.

[17] Baessa M, Rodrigues MJ, Pereira C (2019). A comparative study of the in vitro enzyme inhibitory and antioxidant activities of Butea monosperma (Lam.) Taub. and Sesbania grandiflora (L.) Poiret from Pakistan: new sources of natural products for public health problems. S Afr J Bot.

[18] Das BK, Choudhury BK, Kar M (2010). Quantitative estimation of changes in biochemical constituents of mahua (Madhuca indica syn. Bassia latifolia) flowers during postharvest storage. J Food Process Preserv.

[19] Vinutha K, Pavan G, Pattar S, Kumari NS, Vidya SM (2019). Aqueous extract from Madhuca indica bark protects cells from oxidative stress caused by electron beam radiation: in vitro, in vivo and in silico approach. Heliyon.

[20] Chaudhary A, Bhandari A, Pandurangan A (2012). Antioxidant potential and total phenolic content of methanolic bark extract of Madhuca indica (koenig) Gmelin. Anc Sci Life.

[21] Ryntathiang I, Dharmalingam Jothinathan MK, Behera A, Saravanan S, Murugan R (2024). Comparative bioactivity analysis of green-synthesized metal (cobalt, copper, and selenium) nanoparticles. Cureus.

[22] Behera A, Dharmalingam Jothinathan MK, Saravanan S, Tamil Selvan S, Rajan Renuka R, Srinivasan GP (2024). Green synthesis of Selenium nanoparticles from clove and their toxicity effect and anti-angiogenic, antibacterial and antioxidant potential. Cureus.

[23] Govindasamy R, Raja V, Singh S (2022). Green synthesis and characterization of cobalt oxide nanoparticles using Psidium guajava leaves extracts and their photocatalytic and biological activities. Molecules.

[24] Roat P, Hada S, Chechani B, Yadav DK, Kumar S, Kumari N (2023). Madhuca indica: a review on the phytochemical and pharmacological aspects. Pharm Chem J.

[25] Badukale NA, Panchale WA, Manwar JV, Gudalwar BR, Bakal RL (2021). Phytochemistry, pharmacology and botanical aspects of Madhuca indica: a review. J Pharmacogn Phytochem.

[26] Kumari P, Raina K, Thakur S (2022). Ethnobotany, phytochemistry and pharmacology of palash (Butea monosperma (Lam.) Taub.): a systematic review. Curr Pharmacol Rep.

[27] Polina S, Marka N, Manohar Rao D (2020). Preliminary screening of anti-microbial, anti-oxidant and anti-cancer potential of Butea monosperma flower extracts. Indian J Pure Appl Biosci.

[28] Devidas TB, Vyas A, Sridhar K, Chawla P, Bains A, Sharma M (2023). Valorization of pongame oiltree (Millettia pinnata) seed and seed oil: a promising source of phytochemicals and its applications. Waste Biomass Valorization.

[29] Shabbir MA, Naveed M, Rehman SU (2023). Synthesis of iron oxide nanoparticles from Madhuca indica plant extract and assessment of their cytotoxic, antioxidant, anti-inflammatory, and anti-diabetic properties via different nanoinformatics approaches. ACS Omega.

